# Typhoid Fever among Patients Diagnosed with Dengue in a Tertiary Care Centre: A Descriptive Cross-sectional Study

**DOI:** 10.31729/jnma.7624

**Published:** 2022-08-31

**Authors:** Arun Kumar Mahato, Nischal Shrestha, Sakar Babu Gharti, Madhu Shah

**Affiliations:** 1 Department of Internal Medicine, Nobel Medical College Teaching Hospital, Biratnagar, Morang, Nepal; 2 Department of Pediatrics, Nobel Medical College Teaching Hospital, Biratnagar, Morang, Nepal

**Keywords:** *dengue*, *fever*, *typhoid fever*

## Abstract

**Introduction:**

Dengue and typhoid fever are different entities with overlapping signs and symptoms which are indistinguishable and there have been few reports of co-infections from endemic areas. The resemblance of symptoms makes accurate clinical diagnosis and treatment difficult. Both are major health problems mainly during monsoon and co-infection, if not timely diagnosed and treated can be fatal. The aim of this study was to find out the prevalence of typhoid fever among patients diagnosed with dengue at a tertiary care centre.

**Methods:**

A descriptive cross-sectional study was done among patients of age >15 years with dengue fever attending the medicine outpatient department in a tertiary care centre from 1 July 2021 to 30 June 2022. Ethical approval was taken from the Institutional Review Committee (Reference number: 466/2020). Convenience sampling was used. Patients with other risk factors for febrile illness were excluded from the study. Point estimate and 90% Confidence Interval were calculated.

**Results:**

Among 95 dengue cases, typhoid fever was observed in 18 (18.95%) (12.36-25.54, 90% Confidence Interval). The mean age of presentation was 35±9 years with a male to female ratio of 0.8:1. Fever was the most common presentation with a mean temperature of 100.8±2.1°F.

**Conclusions:**

The prevalence of typhoid fever among dengue-positive cases was higher as compared to other studies done in similar settings.

## INTRODUCTION

Dengue is a viral disease caused by the dengue virus and transmitted by the infective bite of *Aedes aegypti* mosquitoes whereas, typhoid fever is a bacterial disease caused by gram-negative motile bacilli named *Salmonella typhi* and/or *Salmonella para-typhi* and transmitted orally through contaminated food and water.^1,2^

Dengue and typhoid fever are different entities with overlapping signs and symptoms which are indistinguishable and there have been few reports of co-infections from endemic areas in Nepal.^3^ The resemblance of symptoms makes accurate clinical diagnosis and treatment difficult. Both are major health problems in Nepal during monsoon and co-infection, if not timely diagnosed and treated can be fatal.

The aim of this study was to find out the prevalence of typhoid fever among patients diagnosed with dengue at a tertiary care hospital.

## METHODS

A descriptive cross-sectional study was carried out in the Department of Medicine, Nobel Medical College Teaching Hospital, Biratnagar, Nepal over a period of one year from 1 July 2021 to 30 June 2022 after taking ethical approval from the Institutional Review Committee (Reference number: 466/2020). All the patients of >15 years of age with positive serological tests for dengue were included in this study after taking informed consent. Patients with other risk factors for febrile illness were excluded from the study. The convenience sampling technique was used and the sample size was calculated using the formula:


$$\begin{array}{ll} \text{n} & ={\text{Z}}^{2}\times \frac{\text{p}\times \text{q}}{{\text{e}}^{\text{2}}}\\ & =1.64^{2}\times \frac{0.50\times 0.50}{0.09^{2}}\\ & =84 \end{array}$$

Where,

n= minimum required sample size
Z= 1.64 at 90% Confidence Interval (CI)
p= prevalence is taken as 50% for maximum sample size calculation
q= 1-p
e= margin of error, 9%

Adding a 10% non-response rate, the calculated sample size was 93. However, a sample size of 95 dengue patients was taken.

Dengue virus infection can be confirmed by lab tests that include detection of the virus, viral nucleic acid, antigens or antibodies, or a combination of these techniques. Dengue antigen non-structural protein 1 (NS1) is detectable in serum within a few hours up to day 5 from the onset of fever. IgM antibody is detectable by day 3-5 after the onset of illness. IgG antibodies appear after the 14^th^ day of infection and persist for life.^4^ The accuracy of NS1 antigen rapid test is considered higher with a sensitivity of 55-82% and specificity of 97-100%.^5^ The sensitivity and specificity of the rapid test for IgM are 59.70% and 40.20% and for IgG are 50.20% and 49.70%.^6^ Typhidot IgG/IgM Rapid test which detects *Salmonella* antibodies is used for early diagnosis of typhoid fever and has a sensitivity of 41.40% and specificity of 56.50%.^7^

The common clinical manifestations at the time of sample collection were fever, nausea, vomiting, malaise, abdominal discomfort, anorexia, diarrhoea and arthralgia.

Tests were conducted for dengue NS1, IgG and IgM on a rapid strip test. Similarly, Rapid tests for IgM and IgG antibodies against *Salmonella* were done. Data were entered and analysed in IBM SPSS Statistics 24.0. Point estimate and 90% CI were calculated.

## RESULTS

Out of 95 dengue patients, typhoid fever was observed in 18 (18.95%) (12.36-25.54, 90% CI). The mean age of presentation of such cases was 35±9 years. Typhoid fever among dengue was common in the age group of 21-40 years of age. A total of 8 (44.44%) were male and 10 (55.56%) were female with male to female ratio of 0.8:1 (Table 1).

**Table 1:** Sociodemographic variables (n= 18).

Age group	Male n (%)	Female n (%)
≤20	-	1 (5.55)
21-40	6 (33.33)	7 (38.88)
≥41	2 (11.11)	2 (11.11)
Total	8 (44.44)	10 (55.55)

**Figure 1:**
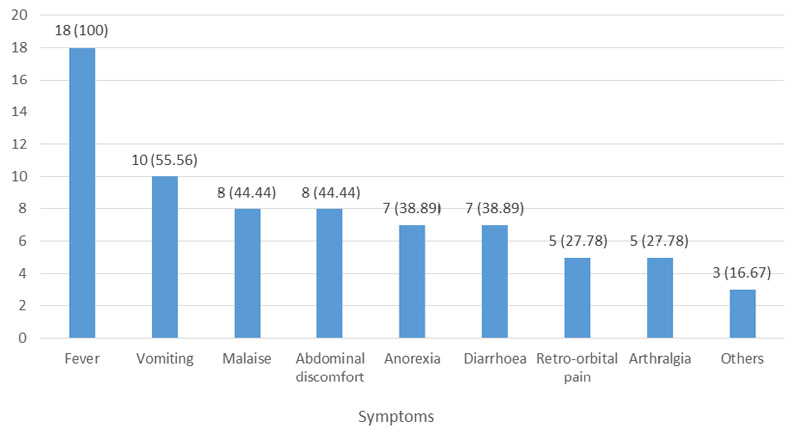
The common presentation of typhoid fever among patients diagnosed with dengue (n= 18).

Fever was the most common symptom observed in 95 (100%) with a mean temperature of 100.8±2.1°F (Figure 1).

The case fatality rate (CFR) was 5.55%. A high dengue IgM with typhoid positivite 16 (88.88%) was noted among patients having typhoid fever diagnosed with dengue, followed by dengue IgG with typhoid 6 (33.33%) and dengue NS1 with typhoid 3 (16.66%) (Table 2).

**Table 2:** Seropositivity of Dengue and typhoid rapid test (n= 18).

Parameters	n (%)
Dengue NS1 with typhoid	3 (16.67)
Dengue IgM with Typhoid	16 (88.89)
Dengue IgG with Typhoid	6 (33.33)
Dengue NS1and IgM with Typhoid	-

## DISCUSSION

The prevalence of typhoid fever among dengue patients in our study was 18.95% which is higher than 7.80% by Sharma,^8^ 6.90% reported by Vimal,^9^ 0.30% by Kasper.^10^ But the prevalence was lesser than 34% as observed in the study done by Naveen.^11^ In this study, male to female ratio was 0.8:1 which is similar to the study done. where female preponderance was noted.^8^ Female gender acquires infection easily during food preparation, other household activities like cleaning with contaminated water and child care. This might have increased the frequency of typhoid fever. Maximum patients were from the age group of 21-40 years. This may be because this is the working age group.

The first case of dengue was reported in Nepal in 2004.^12^ The largest-ever dengue outbreak in Nepal which started in mid-summer was reported in 2019, infecting more than 14,000 people.^13^ Similarly, the annual reports published by the Department of Health Service (DoHS) on typhoid fever show that every year there is an incidence of this disease in every district and its in increasing trend.^14^ The number of reported dengue cases has significantly increased from 3424 in FY 2017/18 to 10808 in F/Y 2018/19. The major cause of increasing the number of reported cases is the impact of the dengue outbreak in Nepal.^15^ The major clinical manifestations of dengue in Nepalese patients reported by Khetan were fever, cerebral pain, rashes, retro-orbital pain, retching, joint pain, and thrombocytopenia.^16^ In dengue fever, there occurs a sharp rise in temperature between 102.2-104°F usually associated with a flushed face, headache mainly retro-orbital, arthralgia, myalgia, and rashes. On the contrary, typhoid fever usually presents with continuous fever of high intensity with a step ladder pattern of increment and usually reaches 104-105.8°F by the end of the first week of illness associated with gastrointestinal symptoms: diarrhoea or constipation.^6,17^

In this study, the mean temperature at presentation was 100.8±2.1°F associated with vomiting, malaise, abdominal discomfort, anorexia, and diarrhoea which is similar to the study by Rajgopal.^18^ Monsoon period is a breeding season for mosquitoes. During this period drinking water also gets contaminated easily. It is well-known fact that bacterial infections follow viral diseases, especially in upper respiratory diseases, and the effect of one disease over the other is not exactly known in dengue-typhoid co-infections.^1,19^ Concurrent infections with more than one etiological agent can result in an illness with overlapping symptoms, resulting in a situation where the diagnosis and management of such a patient could be challenging.^20^ Dengue infection and typhoid fever may overlap, especially during the first few days of illness and are indistinguishable from many other acute febrile illnesses.^21^

All 4 serotypes of Dengue virus (DENV-1, 2, 3 and 4) are found in Nepal.^14^ Once affected by the virus, antibodies that are formed will only prevent re-infection by the same serotype and individuals are susceptible to a second infection with a different serotype so that the risk of dengue hemorrhagic fever and dengue shock syndrome will still be there and co-infection with typhoid fever may accelerate the mortality rate.^11^ Many complications seen are preventable if we can send correct investigation timely, and by monitoring with proper titration of intravenous fluid therapy and correct antibiotics. We can advise patients suspected of infection to take early preventive measures to break the chain of transmission from one to another. The findings of the present study also help the concerned authorities in the endemic areas for early diagnosis and to plan out and implement various preventive and control measures.

This is a single-centre study, the findings of this study cannot be generalised to the whole Nepalese population. There are many endemic areas of dengue and typhoid in Nepal, so the prevalence might be more in those areas.

## CONCLUSIONS

The prevalence of typhoid fever among denguepositive cases was higher as compared to other studies done in similar settings. During monsoon, in endemic areas, we should always be alert keeping in mind the possibility of co-infection in patients presenting with febrile illness. Early diagnosis will prevent the fatal outcome and lowers the patient economic burden.
